# Supplementary material for: Genome-Wide Expression Profile in People with Optic Neuritis Associated with Multiple Sclerosis
Source: Biomedicines. 2023 Aug 7;11(8):2209. doi: 10.3390/biomedicines11082209 (PMC10452153; doi:10.3390/biomedicines11082209)
Supplement: Supplementary file 1 [file biomedicines-11-02209-s001.zip › Supplemental table S1.pdf]

Supplement Data

Table S1. Summary of QC parameters.

Sample	Scaling factor	BG Average	Noise Average	BACT 3'-5' Ratio	GAPDH 3'-5' Ratio
ON1	1.750063	32.768677	1.448748	1.770014	1.385003
ON2	1.405748	35.980556	1.638879	2.095685	1.295887
ON3	2.108592	33.123489	1.538475	1.567522	1.396912
ON4	1.276775	33.362579	1.604414	1.61936	0.941792
ON5	1.194503	33.016277	1.549652	1.953054	1.147624
ON6	1.6843	32.471069	1.468649	1.893243	1.202161
ON7	1.407145	32.737965	1.583432	1.536759	1.14072
ON8	1.632164	35.358902	1.622444	1.695849	1.122931
C1	1.321881	34.56089	1.562648	1.282123	1.098387
C2	1.421009	34.86908	1.506231	1.361225	0.991159
C3	1.733054	32.302567	1.533369	1.807571	1.302708
C4	1.588801	36.027569	1.650865	1.646781	1.238494
C5	1.229317	34.018784	1.887214	1.709209	1.249215
C6	0.807709	38.59164	2.050195	1.338921	0.974054

Overview of major QC parameters showing scaling factors values, average background anise values, as well as 3’-5’ ratios for housekeeping genes Beta-actin (BACT) and glyceraldehyde-3-phosphate dehydrogenase (GAPDH), showing good RNA quality of the analyzed samples.
