# Supplementary material for: Genome-Wide Expression Profile in People with Optic Neuritis Associated with Multiple Sclerosis
Source: Biomedicines. 2023 Aug 7;11(8):2209. doi: 10.3390/biomedicines11082209 (PMC10452153; doi:10.3390/biomedicines11082209)
Supplement: Supplementary file 1 [file biomedicines-11-02209-s001.zip › Supplemental table S2.pdf]

Probe Set ID	Kempinnen Ratio	Keminnen p value	Habek Ratio	Habek p value	Gene Title
228697_at	0,26	0,00	2,22	0,02	histidine triad nucleotide binding protein 3
205624_at	3,80	0,00	0,38	0,00	carboxypeptidase A3 (mast cell)
225239_at	0,70	0,00	2,20	0,02	---
238042_at	1,30	0,00	2,40	0,02	---
1566518_at	0,44	0,00	1,85	0,05	---
206722_s_at	0,44	0,00	2,05	0,04	lysophosphatidic acid receptor 2
227062_at	1,86	0,00	2,42	0,03	non-protein coding RNA 84
208304_at	22,79	0,00	0,40	0,01	chemokine (C-C motif) receptor 3
207067_s_at	2,57	0,00	0,53	0,01	histidine decarboxylase
244677_at	0,61	0,00	1,82	0,02	---
217662_x_at	2,70	0,00	1,83	0,01	---
212438_at	0,84	0,00	0,57	0,00	small nuclear ribonucleoprotein 27kDa (U4/U6.U5)
209710_at	2,62	0,00	0,51	0,01	GATA binding protein 2
207758_at	26,29	0,00	2,05	0,05	hypothetical protein FLJ23185
203300_x_at	0,19	0,00	0,57	0,00	adaptor-related protein complex 1, sigma 2 subunit
1554464_a_at	0,18	0,00	0,59	0,00	cartilage associated protein
231302_at	0,67	0,00	2,44	0,03	---
204426_at	0,61	0,00	0,57	0,03	transmembrane emp24 domain trafficking protein 2
207890_s_at	0,59	0,00	2,00	0,04	matrix metallopeptidase 25
237544_at	0,18	0,00	2,21	0,02	---

223716_s_at	0,12	0,00	0,60	0,04	zinc finger, RAN-binding domain containing 2
209754_s_at	0,76	0,00	0,57	0,03	thymopoietin
235103_at	0,37	0,00	0,53	0,01	mannosidase, alpha, class 2A, member 1
216782_at	4,09	0,00	2,32	0,02	---
238883_at	0,20	0,00	2,76	0,03	---
1568943_at	0,44	0,00	2,33	0,04	inositol polyphosphate-5-phosphatase, 145kDa
244357_at	0,65	0,00	3,46	0,01	---
1558167_a_at	0,56	0,00	0,55	0,00	hypothetical protein MGC16275
223750_s_at	1,98	0,00	1,83	0,05	toll-like receptor 10
243395_at	0,13	0,00	2,27	0,00	---
205076_s_at	0,50	0,00	0,53	0,01	myotubularin related protein 11
236869_at	0,62	0,00	1,96	0,05	---
242352_at	0,94	0,00	1,86	0,03	Nipped-B homolog (Drosophila)
225040_s_at	1,49	0,00	0,60	0,02	ribulose-5-phosphate-3-epimerase
212384_at	31,72	0,00	2,51	0,00	HLA-B associated transcript 1
204285_s_at	0,82	0,00	0,51	0,00	phorbol-12-myristate-13-acetate-induced protein 1
235009_at	7,02	0,00	1,96	0,04	family with sequence similarity 44, member A
226596_x_at	0,89	0,00	0,58	0,01	hypothetical protein LOC729852

214693_x_at	0,36	0,00	1,96	0,02	KIAA1245 /// neuroblastoma breakpoint family, member 1 /// neuroblastoma breakpoint family, member 10 /// neuroblastoma breakpoint family, member 11 /// neuroblastoma breakpoint family, member 14 /// neuroblastoma breakpoint family, member 16 /// neurobla
232225_at	1,68	0,00	2,03	0,01	---
238678_at	0,99	0,00	3,57	0,04	hypothetical LOC402483
237591_at	0,38	0,00	1,85	0,02	FLJ42957 protein
223940_x_at	0,60	0,00	2,17	0,04	metastasis associated lung adenocarcinoma transcript 1 (non-protein coding)
1563509_at	0,08	0,00	1,87	0,02	---
222507_s_at	0,69	0,00	0,59	0,00	TMEM9 domain family, member B
1554565_x_at	3,15	0,00	2,57	0,04	signal peptide peptidase 3
200999_s_at	3,14	0,00	1,84	0,03	cytoskeleton-associated protein 4
204713_s_at	1,35	0,00	2,09	0,04	coagulation factor V (proaccelerin, labile factor)

224568_x_at	18,11	0,00	2,40	0,02	metastasis associated lung adenocarcinoma transcript 1 (non-protein coding)
219906_at	0,43	0,00	1,95	0,01	hypothetical protein FLJ10213
242449_at	2,26	0,00	1,90	0,05	---
202783_at	1,24	0,00	0,57	0,00	nicotinamide nucleotide transhydrogenase
220712_at	0,83	0,00	2,43	0,05	chromosome 8 open reading frame 60
229457_at	1,47	0,00	2,28	0,04	ankyrin repeat and KH domain containing 1
235390_at	0,42	0,00	0,52	0,00	SFRS12-interacting protein 1
235536_at	0,17	0,00	1,84	0,03	small nucleolar RNA, C/D box 89
230707_at	0,42	0,00	1,98	0,02	sortilin-related receptor, L(DLR class) A repeats-containing
224919_at	1,36	0,00	0,57	0,00	mitochondrial ribosomal protein S6
214765_s_at	0,33	0,00	0,60	0,00	N-acylethanolamine acid amidase
211947_s_at	88,21	0,00	3,16	0,01	BAT2 domain containing 1
1569422_at	0,68	0,00	3,85	0,04	family with sequence similarity 129, member C
234210_x_at	0,06	0,00	2,24	0,04	Actin-related protein Arp2 (ARP2)

206792_x_at	0,07	0,00	1,89	0,02	phosphodiesterase 4C, cAMP-specific (phosphodiesterase E1 dunce homolog, Drosophila)
218338_at	1,81	0,00	0,59	0,00	polyhomeotic homolog 1 (Drosophila) /// polyhomeotic homolog 1B (Drosophila)
242776_at	0,20	0,00	1,84	0,05	zinc finger, CCHC domain containing 6
230180_at	0,83	0,00	2,35	0,03	---
200084_at	2,73	0,00	0,60	0,01	chromosome 11 open reading frame 58
202505_at	0,23	0,00	0,51	0,00	small nuclear ribonucleoprotein polypeptide B''
235346_at	0,42	0,00	0,60	0,00	FUN14 domain containing 1
215147_at	1,90	0,00	1,96	0,03	---
202124_s_at	2,19	0,00	0,59	0,01	trafficking protein, kinesin binding 2
223259_at	1,32	0,00	0,57	0,01	ORM1-like 3 (S. cerevisiae)
239143_x_at	0,29	0,00	0,55	0,04	ring finger protein 138
226962_at	0,90	0,00	0,55	0,02	zinc finger and BTB domain containing 41
220246_at	0,87	0,00	1,95	0,01	calcium/calmodulin-dependent protein kinase ID
235170_at	0,32	0,00	0,51	0,02	zinc finger protein 92
231222_at	3,09	0,00	2,36	0,05	---

230341_x_at	0,47	0,00	0,57	0,02	ADAM metallopeptidase with thrombospondin type 1 motif, 10
222668_at	3,05	0,00	0,57	0,02	potassium channel tetramerisation domain containing 15
213183_s_at	7,50	0,00	0,32	0,02	Cyclin-dependent kinase inhibitor 1C transcript variant 3 (CDKN1C) mRNA, complete cds, alternatively spliced
205308_at	1,53	0,00	0,39	0,01	family with sequence similarity 164, member A
229317_at	0,50	0,00	0,49	0,00	karyopherin alpha 5 (importin alpha 6)
242688_at	0,35	0,00	2,68	0,04	---
203787_at	0,98	0,00	0,56	0,00	single-stranded DNA binding protein 2
240458_at	0,30	0,00	1,82	0,01	---
234044_at	5,15	0,00	3,27	0,02	---
222907_x_at	0,34	0,00	0,59	0,01	transmembrane protein 50B
234151_at	0,18	0,00	2,02	0,02	---
205885_s_at	0,28	0,00	0,57	0,02	integrin, alpha 4 (antigen CD49D, alpha 4 subunit of VLA-4 receptor)

208451_s_at	1,16	0,00	0,53	0,02	complement component 4A (Rodgers blood group) /// complement component 4B (Chido blood group)
203020_at	1,36	0,00	0,55	0,00	RAB GTPase activating protein 1-like
211968_s_at	1,43	0,00	0,59	0,00	heat shock protein 90kDa alpha (cytosolic), class A member 1
229367_s_at	0,13	0,00	0,59	0,00	GTPase, IMAP family member 6
1552480_s_at	2,74	0,00	4,32	0,01	protein tyrosine phosphatase, receptor type, C
224722_at	4,91	0,00	0,53	0,04	mindbomb homolog 1 (Drosophila)
235327_x_at	0,12	0,00	2,57	0,04	UBX domain protein 2A
201017_at	5,09	0,00	0,56	0,03	eukaryotic translation initiation factor 1A, X-linked
216401_x_at	0,17	0,00	0,54	0,03	similar to Ig kappa chain V-I region HK102 precursor
240248_at	5,57	0,00	2,03	0,02	---
234326_at	1,33	0,00	2,71	0,01	---
226051_at	2,09	0,00	0,56	0,01	selenoprotein M
220023_at	0,84	0,00	2,07	0,01	apolipoprotein B48 receptor
232472_at	0,41	0,00	2,45	0,04	---
201833_at	2,29	0,00	0,59	0,00	histone deacetylase 2
1556204_a_at	4,05	0,00	4,62	0,01	Full length insert cDNA clone ZE16C11

243860_at	0,92	0,00	2,58	0,04	---
220809_at	1,69	0,00	2,98	0,04	hypothetical protein FLJ14327
236199_at	0,99	0,00	1,88	0,03	---
232551_at	0,32	0,00	2,07	0,03	solute carrier family 26, member 6
206877_at	0,98	0,00	4,02	0,00	MAX dimerization protein 1
1566608_at	0,08	0,00	2,06	0,04	---
213146_at	5,52	0,00	1,89	0,03	jumonji domain containing 3, histone lysine demethylase
204137_at	0,69	0,00	0,57	0,01	G protein-coupled receptor 137B
234886_at	0,26	0,00	0,50	0,00	T cell receptor beta variable 24-1
215628_x_at	0,80	0,00	1,84	0,00	---
211305_x_at	6,40	0,00	1,86	0,03	Fc fragment of IgA, receptor for
237475_x_at	0,06	0,00	2,93	0,04	coiled-coil domain containing 152
203347_s_at	0,84	0,00	0,60	0,00	metal response element binding transcription factor 2
208146_s_at	0,07	0,00	0,53	0,00	carboxypeptidase, vitellogenic-like
244808_at	0,44	0,01	2,19	0,03	GRAM domain containing 1A, mRNA (cDNA clone IMAGE:5921205)
229389_at	0,54	0,01	2,24	0,02	ATG16 autophagy related 16-like 2 (S. cerevisiae)
222371_at	0,66	0,01	2,21	0,03	---

203386_at	1,36	0,01	0,44	0,00	TBC1 domain family, member 4
1564378_a_at	1,40	0,01	2,38	0,01	---
211050_x_at	37,77	0,01	2,23	0,04	similar to hypothetical protein LOC284701 /// similar to hypothetical protein LOC284701
209696_at	0,38	0,01	0,52	0,00	fructose-1,6-bisphosphatase 1
216859_x_at	0,43	0,01	1,82	0,04	---
243554_at	1,00	0,01	2,13	0,05	---
209123_at	4,86	0,01	0,49	0,00	quinoid dihydropteridine reductase
211316_x_at	0,42	0,01	2,04	0,02	CASP8 and FADD-like apoptosis regulator
204026_s_at	3,64	0,01	0,59	0,03	ZW10 interactor
205884_at	0,39	0,01	0,52	0,01	integrin, alpha 4 (antigen CD49D, alpha 4 subunit of VLA-4 receptor)
204612_at	4,24	0,01	0,54	0,00	protein kinase (cAMP-dependent, catalytic) inhibitor alpha
232179_at	2,38	0,01	2,88	0,03	---
239661_at	1,34	0,01	2,29	0,01	---
241722_x_at	0,20	0,01	1,84	0,01	---
213534_s_at	1,23	0,01	0,52	0,00	PAS domain containing serine/threonine kinase
235841_at	3,00	0,01	2,39	0,02	---
212611_at	5,35	0,01	0,60	0,03	deltex 4 homolog (Drosophila)

204127_at	1,20	0,01	0,58	0,02	replication factor C (activator 1) 3, 38kDa
205771_s_at	1,20	0,01	0,58	0,00	A kinase (PRKA) anchor protein 7
219342_at	0,93	0,01	0,59	0,03	CAS1 domain containing 1
1558732_at	0,98	0,01	1,80	0,01	mitogen-activated protein kinase kinase kinase kinase 4
234621_at	1,86	0,01	2,69	0,01	---
222336_at	1,51	0,01	2,47	0,04	chromosome 4 open reading frame 34
230505_at	1,45	0,01	1,97	0,05	hypothetical protein LOC145474
229483_at	0,36	0,01	1,89	0,01	---
216858_x_at	0,17	0,01	3,19	0,05	---
242827_x_at	0,25	0,01	2,16	0,03	---
240665_at	0,08	0,01	2,13	0,04	---
242163_at	3,54	0,01	2,53	0,02	thyroid hormone receptor associated protein 3
207435_s_at	0,19	0,01	1,82	0,03	serine/arginine repetitive matrix 2
206682_at	0,65	0,01	0,56	0,01	C-type lectin domain family 10, member A
243954_at	1,76	0,01	1,89	0,02	hypothetical protein LOC285286
212944_at	0,94	0,01	0,57	0,01	solute carrier family 5 (sodium/myo-inositol cotransporter), member 3
1557852_at	0,66	0,01	5,07	0,05	---
204112_s_at	0,46	0,01	0,57	0,04	histamine N-methyltransferase

226425_at	0,51	0,01	0,59	0,04	CAP-GLY domain containing linker protein family, member 4
235046_at	0,26	0,01	0,57	0,03	---
204460_s_at	1,73	0,01	0,60	0,00	RAD1 homolog (S. pombe)
212468_at	4,68	0,01	1,85	0,00	sperm associated antigen 9
213864_s_at	0,43	0,01	0,52	0,00	nucleosome assembly protein 1-like 1
217188_s_at	0,53	0,01	0,55	0,02	chromosome 14 open reading frame 1
229445_at	4,27	0,01	2,07	0,00	cytochrome b-245, alpha polypeptide
237875_at	0,56	0,01	1,93	0,00	---
1556762_a_at	1,62	0,01	2,14	0,02	---
240498_at	0,43	0,01	2,25	0,01	---
236645_at	1,58	0,01	2,08	0,04	HMG-box transcription factor 1
1565714_at	6,20	0,01	1,96	0,01	---
229871_at	0,77	0,01	1,81	0,04	sterile alpha motif domain containing 4B
224413_s_at	0,34	0,01	0,58	0,00	TM2 domain containing 2
244548_at	0,20	0,01	2,65	0,02	---
244871_s_at	0,55	0,01	2,20	0,01	ubiquitin specific peptidase 32
1559449_a_at	1,73	0,01	2,32	0,00	CDNA FLJ58216 complete cds, highly similar to Zinc finger protein 539
1566549_at	0,60	0,01	2,22	0,02	---
244423_at	6,25	0,01	2,81	0,02	---
238044_at	1,73	0,01	1,84	0,03	---
242407_at	0,42	0,01	2,25	0,04	---
223361_at	0,13	0,01	0,52	0,00	chromosome 6 open reading frame 115

213212_x_at	3,58	0,01	0,56	0,02	golgi autoantigen, golgin subfamily a-like pseudogene /// hypothetical protein LOC440295 /// hypothetical LOC643696 /// similar to golgi autoantigen, golgin subfamily a-like
201625_s_at	4,87	0,02	0,56	0,00	insulin induced gene 1
227454_at	1,04	0,02	1,83	0,00	TAO kinase 1
243578_at	2,32	0,02	2,28	0,01	---
218864_at	21,22	0,02	0,55	0,02	tensin 1
240870_at	0,99	0,02	3,22	0,01	---
244840_x_at	2,85	0,02	2,38	0,00	dedicator of cytokinesis 4
244556_at	0,23	0,02	2,11	0,01	CDNA FLJ57378 complete cds, highly similar to Lymphocyte cytosolic protein 2
203156_at	0,34	0,02	0,57	0,01	A kinase (PRKA) anchor protein 11
210504_at	8,98	0,02	0,54	0,01	Kruppel-like factor 1 (erythroid)
224517_at	0,29	0,02	1,85	0,04	polymerase (RNA) II (DNA directed) polypeptide J4, pseudogene
229364_at	4,58	0,02	2,16	0,04	hypothetical protein LOC646870
217144_at	0,44	0,02	0,60	0,04	similar to ubiquitin B /// ribosomal protein S27a /// ubiquitin B /// ubiquitin C
223240_at	2,42	0,02	0,57	0,03	F-box protein 8
238544_at	12,45	0,02	3,79	0,02	---

218929_at	0,60	0,02	0,59	0,01	CDKN2A interacting protein
1562957_at	0,46	0,02	2,51	0,00	---
204244_s_at	0,87	0,02	0,57	0,01	DBF4 homolog (S. cerevisiae)
1562063_x_at	0,08	0,02	1,82	0,04	KIAA1245 /// neuroblastoma breakpoint family, member 1 /// neuroblastoma breakpoint family, member 10 /// neuroblastoma breakpoint family, member 11 /// neuroblastoma breakpoint family, member 14 /// neuroblastoma breakpoint family, member 16 /// neurobla
239629_at	0,06	0,02	2,39	0,05	I-FLICE isoform 5
207194_s_at	1,40	0,02	0,51	0,02	intercellular adhesion molecule 4 (Landsteiner-Wiener blood group)
207198_s_at	0,11	0,02	0,59	0,00	LIM and senescent cell antigen-like domains 1
236243_at	0,88	0,02	1,99	0,02	MRNA; cDNA DKFZp666B142 (from clone DKFZp666B142)
223662_x_at	0,26	0,02	1,91	0,05	DEAD (Asp-Glu-Ala-Asp) box polypeptide 59
209457_at	6,72	0,02	0,59	0,03	dual specificity phosphatase 5

221268_s_at	0,60	0,02	0,58	0,02	sphingosine-1-phosphate phosphatase 1
220748_s_at	1,63	0,02	1,92	0,01	zinc finger protein 580
204141_at	2,42	0,02	0,45	0,04	tubulin, beta 2A
233427_x_at	0,17	0,02	1,96	0,02	---
1559882_at	0,18	0,02	3,00	0,03	Full length insert cDNA clone YP80A10
1554501_at	0,44	0,02	1,94	0,04	TSC22 domain family, member 4
234970_at	2,02	0,02	0,59	0,02	tandem C2 domains, nuclear
218967_s_at	0,48	0,02	0,58	0,00	phosphotriesterase related
230389_at	0,50	0,03	1,88	0,05	formin binding protein 1
237403_at	0,40	0,03	0,52	0,01	growth factor independent 1B transcription repressor
202203_s_at	4,04	0,03	0,58	0,01	autocrine motility factor receptor
222588_s_at	0,94	0,03	0,58	0,01	chromosome 11 open reading frame 57
232058_at	0,55	0,03	2,85	0,05	---
241403_at	1,07	0,03	2,71	0,01	CDC-like kinase 4
219077_s_at	1,19	0,03	0,55	0,01	WW domain containing oxidoreductase
233315_at	0,76	0,03	2,77	0,02	---
53912_at	1,14	0,03	0,60	0,01	sorting nexin 11
1568915_at	0,87	0,03	2,05	0,00	---
238560_at	0,80	0,03	1,90	0,05	calcium binding and coiled-coil domain 2
205094_at	1,61	0,03	0,57	0,00	peroxisomal biogenesis factor 12

203387_s_at	1,45	0,03	0,49	0,00	TBC1 domain family, member 4
210895_s_at	0,50	0,03	0,58	0,02	CD86 molecule
221710_x_at	10,95	0,03	0,47	0,00	family with sequence similarity 176, member B /// hypothetical protein LOC100133999
216593_s_at	0,15	0,03	0,54	0,00	phosphatidylinositol glycan anchor biosynthesis, class C
232658_at	1,94	0,03	2,17	0,04	---
1555243_x_at	0,39	0,03	1,88	0,02	chromosome 8 open reading frame 59
203593_at	1,02	0,03	0,55	0,01	CD2-associated protein
211768_at	15,20	0,03	2,19	0,02	linker for activation of T cells family, member 2
244433_at	0,12	0,03	2,22	0,05	---
1556055_at	0,67	0,03	1,90	0,02	---
242279_at	3,23	0,03	1,90	0,00	---
235179_at	1,52	0,03	1,97	0,05	zinc finger protein 641
243117_at	2,35	0,03	1,86	0,03	---
210679_x_at	0,39	0,04	2,86	0,05	---
228770_at	4,89	0,04	0,59	0,03	G protein-coupled receptor 146
227935_s_at	0,37	0,04	0,57	0,02	polycomb group ring finger 5
239379_at	0,40	0,04	2,01	0,04	LRR FLI-I interacting protein 1 (LRRFIP1)
233816_at	1,16	0,04	4,11	0,04	---

1558792_x_at	0,49	0,04	3,13	0,04	CDNA FLJ61453 complete cds, highly similar to Adapter-relatedprotein complex 2 alpha-1 subunit
213624_at	1,09	0,04	0,50	0,01	sphingomyelin phosphodiesterase, acid-like 3A
220532_s_at	0,06	0,04	0,44	0,00	transmembrane protein 176B
214188_at	2,67	0,04	2,24	0,01	---
242642_at	3,24	0,04	2,51	0,01	---
219029_at	1,26	0,04	0,55	0,05	chromosome 5 open reading frame 28
1560230_at	2,02	0,04	2,91	0,05	---
1566964_at	2,16	0,04	4,46	0,04	---
209841_s_at	1,25	0,04	0,53	0,05	leucine rich repeat neuronal 3
240279_at	0,70	0,04	1,85	0,01	---
218793_s_at	2,39	0,04	0,57	0,04	sex comb on midleg-like 1 (Drosophila)
212671_s_at	0,17	0,04	0,56	0,03	major histocompatibility complex, class II, DQ alpha 1 /// major histocompatibility complex, class II, DQ alpha 2
206488_s_at	0,09	0,04	0,54	0,01	CD36 molecule (thrombospondin receptor)
1561015_at	0,25	0,04	1,94	0,04	---
200644_at	4,76	0,04	0,55	0,01	MARCKS-like 1
243537_at	0,39	0,04	2,61	0,03	---
218983_at	0,94	0,04	1,93	0,03	complement component 1, r subcomponent-like

222573_s_at	1,19	0,04	0,59	0,02	salvador homolog 1 (Drosophila)
222317_at	0,25	0,04	0,48	0,00	---
230004_at	0,84	0,05	2,08	0,03	ubiquitin specific peptidase 24
222805_at	0,99	0,05	0,55	0,02	mannosidase, endo-alpha
218273_s_at	1,20	0,05	0,53	0,00	protein phosphatase 2C, magnesium-dependent, catalytic subunit
226783_at	1,08	0,05	1,93	0,03	alanine-glyoxylate aminotransferase 2-like 2
212061_at	2,84	0,05	0,60	0,02	U2-associated SR140 protein
243874_at	0,58	0,05	2,89	0,05	Lipoma cell line Li-14/SV40 ectopic sequence from HMGI-C fusion mRNA, 3' sequence, clone pCH108
226856_at	5,99	0,05	2,36	0,02	musculoskeletal, embryonic nuclear protein 1
225290_at	0,42	0,05	0,54	0,02	ethanolamine kinase 1
214989_x_at	0,69	0,05	2,02	0,01	---
224740_at	0,73	0,05	0,53	0,00	chromosome 5 open reading frame 43
227114_at	2,01	0,05	0,59	0,01	ring finger protein 214
213853_at	5,21	0,05	0,60	0,05	DnaJ (Hsp40) homolog, subfamily C, member 24
236802_at	1,50	0,05	2,85	0,02	---
236931_at	0,31	0,05	2,73	0,02	---

233371_at	5,68	0,05	0,52	0,02	ATP-binding cassette, sub-family C (CFTR/MRP), member 13
206363_at	1,52	0,05	0,54	0,03	v-maf musculoaponeurotic fibrosarcoma oncogene homolog (avian)
232784_at	0,72	0,05	2,81	0,04	---
215797_at	4,12	0,05	0,52	0,01	T cell receptor alpha variable 8-3
231332_at	0,28	0,05	2,07	0,03	---

Gene Symbol
HINT3
CPA3
LPAR2
NCRNA00084
CCR3
HDC
SNRNP27
GATA2
FLJ23185
AP1S2
CRTAP
TMED2
MMP25

ZRANB2
TMPO
MAN2A1

INPP5D

MGC16275
TLR10

MTMR11

NIPBL
RPE
BAT1
PMAIP1
FAM44A
tcag7.903

KIAA1245 /// NBPF1 /// NBPF10 /// NBPF11 /// NBPF14 /// NBPF16 /// NBPF20 /// NBPF8 /// NBPF9 /// RP11-94I2.2
---
tcag7.907
FLJ42957
MALAT1

TMEM9B
UNQ1887
CKAP4
F5

MALAT1
FLJ10213

NNT
C8orf60
ANKHD1
SFRS12IP1
SNORD89
SORL1
MRPS6
NAAA
BAT2D1
FAM129C
ACTR2

PDE4C
PHC1 /// PHC1B
ZCCHC6

C11orf58
SNRPB2
FUNDC1

TRAK2
ORMDL3
RNF138
ZBTB41
CAMK1D
ZNF92

ADAMTS10
KCTD15
CDKN1C
FAM164A
KPNA5

SSBP2

TMEM50B

ITGA4

C4A /// C4B
RABGAP1L
HSP90AA1
GIMAP6
PTPRC
MIB1
UBXN2A
EIF1AX
LOC652493

SELM
APOB48R

HDAC2
LOC730051

FLJ14327
SLC26A6
MXD1
JMJD3
GPR137B
TRBV24-1
FCAR
CCDC152
MTF2
CPVL
GRAMD1A
ATG16L2

TBC1D4

DKFZP434B2016 /// LOC643313
FBP1

QDPR
CFLAR
ZWINT
ITGA4
PKIA

PASK

DTX4

RFC3
AKAP7
CASD1
MAP4K4

C4orf34
LOC145474

THRAP3
SRRM2
CLEC10A
LOC285286
SLC5A3

HNMT

CLIP4

RAD1
SPAG9
NAP1L1
C14orf1
CYBA

HBP1

SAMD4B
TM2D2

USP32
ZNF254

C6orf115

FLJ40113 /// LOC440295 /// LOC643696 /// LOC728636
INSIG1
TAOK1

TNS1

DOCK4
LCP2
AKAP11
KLF1
POLR2J4
LOC646870
LOC648390 /// RPS27A /// UBB /// UBC
FBXO8

CDKN2AIP

DBF4
KIAA1245 /// NBPF1 /// NBPF10 /// NBPF11 /// NBPF14 /// NBPF16 /// NBPF20 /// NBPF3 /// NBPF8 /// NBPF9 /// RP11-94I2.2
CFLAR
ICAM4
LIMS1
ZCCHC6
DDX59
DUSP5

SGPP1
ZNF580
TUBB2A

SAMHD1
TSC22D4
TC2N
PTER
FNBP1
GFI1B
AMFR
C11orf57

CLK4
WWOX

SNX11

CALCOCO2
PEX12

TBC1D4
CD86
FAM176B /// LOC100133999
PIGC

C8orf59
CD2AP
LAT2

ZNF641

GPR146
PCGF5
LRRFIP1

AP2A1
SMPDL3A
TMEM176B

C5orf28

LRRN3

SCML1
HLA-DQA1 /// HLA-DQA2
CD36

MARCKSL1

C1RL

SAV1
USP24
MANEA
PPM2C
AGXT2L2
SR140
LPP
MUSTN1
ETNK1
C5orf43
RNF214
DNAJC24

ABCC13
MAF

TRAV8-3
