# Supplementary material for: Genome-Wide Expression Profile in People with Optic Neuritis Associated with Multiple Sclerosis
Source: Biomedicines. 2023 Aug 7;11(8):2209. doi: 10.3390/biomedicines11082209 (PMC10452153; doi:10.3390/biomedicines11082209)
Supplement: Supplementary file 1 [file biomedicines-11-02209-s001.zip › Supplemental table S3.pdf]

NAME	PROBE	GENE SYM	GENE_TITLE	RANK IN G	RANK MET	RUNNING	CORE ENRICHMENT
1	NFKBIA	NFKBIA	nuclear factor of kappa light polypeptide gene enhancer in B-cells inhibitor, alpha	516	0,772533	0,06895	Yes
2	IL1B	IL1B	interleukin 1, beta	598	0,742461	0,155371	Yes
3	DUSP1	DUSP1	dual specificity phosphatase 1	601	0,74201	0,245576	Yes
4	TLR2	TLR2	toll-like receptor 2	693	0,714503	0,328109	Yes
5	MAPK14	MAPK14	mitogen-activated protein kinase 14	1782	0,511492	0,337503	Yes
6	CREBBP	CREBBP	CREB binding protein (Rubinstein-Taybi syndrome)	1840	0,503742	0,396039	Yes
7	MYD88	MYD88	myeloid differentiation primary response gene (88)	2502	0,423297	0,415443	Yes
8	EP300	EP300	E1A binding protein p300	2539	0,419031	0,46469	Yes
9	MAP2K6	MAP2K6	mitogen-activated protein kinase kinase 6	2687	0,402073	0,50648	Yes
10	NFKB1	NFKB1	nuclear factor of kappa light polypeptide gene enhancer in B-cells 1 (p105)	2828	0,38922	0,547047	Yes
11	RELA	RELA	v-rel reticuloendotheliosis viral oncogene homolog A, nuclear factor of kappa light polypeptide gene enhancer in B-cells 3, p65 (avian)	3086	0,364392	0,578908	Yes
12	CHUK	CHUK	conserved helix-loop-helix ubiquitous kinase	4090	0,276936	0,563886	Yes
13	TGFBR2	TGFBR2	transforming growth factor, beta receptor II (70/80kDa)	4099	0,276465	0,597143	Yes
14	NR3C1	NR3C1	nuclear receptor subfamily 3, group C, member 1 (glucocorticoid receptor)	4148	0,272569	0,627982	Yes
15	TGFBR1	TGFBR1	transforming growth factor, beta receptor I (activin A receptor type II-like kinase, 53kDa)	5433	0,177547	0,587214	No
16	IL8	IL8	interleukin 8	5994	0,140218	0,577074	No
17	MAPK11	MAPK11	mitogen-activated protein kinase 11	7902	0,025589	0,487548	No
18	MAP3K7	MAP3K7	mitogen-activated protein kinase kinase kinase 7	12280	-0,19735	0,298934	No
19	IKBKB	IKBKB	inhibitor of kappa light polypeptide gene enhancer in B-cells, kinase beta	12376	-0,20193	0,318894	No
20	TNF	TNF	tumor necrosis factor (TNF superfamily, member 2)	13227	-0,24637	0,307584	No
21	MAP3K14	MAP3K14	mitogen-activated protein kinase kinase kinase 14	16260	-0,41729	0,211076	No
