# Supplementary material for: Genome-Wide Expression Profile in People with Optic Neuritis Associated with Multiple Sclerosis
Source: Biomedicines. 2023 Aug 7;11(8):2209. doi: 10.3390/biomedicines11082209 (PMC10452153; doi:10.3390/biomedicines11082209)
Supplement: Supplementary file 1 [file biomedicines-11-02209-s001.zip › Supplemental table S4.pdf]

NAME	PROBE	GENE SYM	GENE_TITLE	RANK IN G	RANK MET	RUNNING	CORE ENRICHMENT
1	IL13RA1	IL13RA1	interleukin 13 receptor, alpha 1	280	0,897462	0,079823	Yes
1	JAK3	JAK3	Janus kinase 3 (a protein tyrosine kinase, leukocyte)	364	0,839101	0,163142	Yes
1	SOS2	SOS2	son of sevenless homolog 2 (Drosophila)	380	0,830956	0,248918	Yes
1	INPP5D	INPP5D	inositol polyphosphate-5-phosphatase, 145kDa	686	0,715864	0,308622	Yes
1	NR0B2	NR0B2	nuclear receptor subfamily 0, group B, member 2	1012	0,641317	0,359592	Yes
1	IL4R	IL4R	interleukin 4 receptor	1419	0,565144	0,398698	Yes
1	GRB2	GRB2	growth factor receptor-bound protein 2	1802	0,50902	0,433126	Yes
1	CISH	CISH	cytokine inducible SH2-containing protein	1883	0,497381	0,481018	Yes
1	PI3	PI3	peptidase inhibitor 3, skin-derived (SKALP)	2007	0,481655	0,525183	Yes
1	IL2RG	IL2RG	interleukin 2 receptor, gamma (severe combined immunodeficiency)	2644	0,408205	0,536774	Yes
1	AKT1	AKT1	v-akt murine thymoma viral oncogene homolog 1	3391	0,337864	0,535698	Yes
1	STAT6	STAT6	signal transducer and activator of transcription 6, interleukin-4 induced	4317	0,260514	0,517872	Yes
1	TYK2	TYK2	tyrosine kinase 2	4344	0,258741	0,543544	Yes
1	SHC1	SHC1	SHC (Src homology 2 domain containing) transforming protein 1	4989	0,207715	0,533875	Yes
1	JAK2	JAK2	Janus kinase 2 (a protein tyrosine kinase)	5165	0,196435	0,545821	Yes
1	JAK1	JAK1	Janus kinase 1 (a protein tyrosine kinase)	6938	0,083244	0,468384	No
1	PPP1R13B	PPP1R13B	protein phosphatase 1, regulatory (inhibitor) subunit 13B	6985	0,080474	0,474526	No
1	PIK3CA	PIK3CA	phosphoinositide-3-kinase, catalytic, alpha polypeptide	8732	-0,01801	0,391562	No
1	AKT3	AKT3	v-akt murine thymoma viral oncogene homolog 3 (protein kinase B, gamma)	9855	-0,07885	0,345251	No
1	IL4	IL4	interleukin 4	9970	-0,08439	0,348497	No
1	AKT2	AKT2	v-akt murine thymoma viral oncogene homolog 2	10992	-0,1342	0,312857	No
1	SERPINA4	SERPINA4	serpin peptidase inhibitor, clade A (alpha-1 antiproteinase, antitrypsin), member 4	11003	-0,13503	0,326428	No
1	RPS6KB1	RPS6KB1	ribosomal protein S6 kinase, 70kDa, polypeptide 1	11440	-0,15742	0,32163	No
1	SOS1	SOS1	son of sevenless homolog 1 (Drosophila)	12849	-0,22749	0,276897	No
1	SRC	SRC	v-src sarcoma (Schmidt-Ruppin A-2) viral oncogene homolog (avian)	16364	-0,42409	0,150298	No
1	IARS	IARS	isoleucine-tRNA synthetase	17840	-0,53531	0,134354	No
