# Supplementary material for: Genome-Wide Expression Profile in People with Optic Neuritis Associated with Multiple Sclerosis
Source: Biomedicines. 2023 Aug 7;11(8):2209. doi: 10.3390/biomedicines11082209 (PMC10452153; doi:10.3390/biomedicines11082209)
Supplement: Supplementary file 1 [file biomedicines-11-02209-s001.zip › Supplemental table S5.pdf]

NAME	PROBE	GENE SYM	GENE_TITLE	RANK IN G	RANK MET	RUNNING	CORE ENRICHMENT
1	JAK3	JAK3	Janus kinase 3 (a protein tyrosine kinase, leukocyte)	364	0,839101	0,073302	Yes
2	SOS2	SOS2	son of sevenless homolog 2 (Drosophila)	380	0,830956	0,162679	Yes
3	PIK3CD	PIK3CD	phosphoinositide-3-kinase, catalytic, delta polypeptide	837	0,678505	0,214095	Yes
4	IRS2	IRS2	insulin receptor substrate 2	1290	0,588575	0,255954	Yes
5	IL4R	IL4R	interleukin 4 receptor	1419	0,565144	0,311017	Yes
6	RAF1	RAF1	v-raf-1 murine leukemia viral oncogene homolog 1	1549	0,547035	0,364067	Yes
7	GRB2	GRB2	growth factor receptor-bound protein 2	1802	0,50902	0,407018	Yes
8	GSK3A	GSK3A	glycogen synthase kinase 3 alpha	1925	0,49251	0,454496	Yes
9	BCL2	BCL2	B-cell CLL/lymphoma 2	2062	0,47321	0,499201	Yes
10	SOCS1	SOCS1	suppressor of cytokine signaling 1	2173	0,459333	0,543664	Yes
11	MAPK3	MAPK3	mitogen-activated protein kinase 3	2605	0,411976	0,567394	Yes
12	MAPK1	MAPK1	mitogen-activated protein kinase 1	3351	0,341823	0,568258	Yes
13	AKT1	AKT1	v-akt murine thymoma viral oncogene homolog 1	3391	0,337864	0,603	Yes
14	GSK3B	GSK3B	glycogen synthase kinase 3 beta	3896	0,29226	0,610201	Yes
15	STAT6	STAT6	signal transducer and activator of transcription 6, interleukin-4 induced	4317	0,260514	0,618041	Yes
16	SHC1	SHC1	SHC (Src homology 2 domain containing) transforming protein 1	4989	0,207715	0,607959	No
17	JAK1	JAK1	Janus kinase 1 (a protein tyrosine kinase)	6938	0,083244	0,522326	No
18	PPP1R13B	PPP1R13B	protein phosphatase 1, regulatory (inhibitor) subunit 13B	6985	0,080474	0,528817	No
19	MAP4K1	MAP4K1	mitogen-activated protein kinase kinase kinase kinase 1	7528	0,048716	0,507762	No
20	PIK3CA	PIK3CA	phosphoinositide-3-kinase, catalytic, alpha polypeptide	8732	-0,01801	0,451257	No
21	PDK1	PDK1	pyruvate dehydrogenase kinase, isozyme 1	9297	-0,04927	0,429193	No
22	AKT3	AKT3	v-akt murine thymoma viral oncogene homolog 3 (protein kinase B, gamma)	9855	-0,07885	0,410677	No
23	AKT2	AKT2	v-akt murine thymoma viral oncogene homolog 2	10992	-0,1342	0,370028	No
24	BAD	BAD	BCL2-antagonist of cell death	11536	-0,16143	0,361147	No
25	PIK3R1	PIK3R1	phosphoinositide-3-kinase, regulatory subunit 1 (p85 alpha)	12013	-0,18356	0,357921	No
26	SOS1	SOS1	son of sevenless homolog 1 (Drosophila)	12849	-0,22749	0,342015	No
27	IRS1	IRS1	insulin receptor substrate 1	14625	-0,32117	0,290588	No
